# Targeting Hdm2 and Hdm4 in Anticancer Drug Discovery: Implications for Checkpoint Inhibitor Immunotherapy

**DOI:** 10.3390/cells13131124

**Published:** 2024-06-29

**Authors:** Monde Ntwasa

**Affiliations:** Department of Life and Consumer Sciences, University of South Africa, Cnr Pioneer Road and Christiaan de Wet Road, Florida, Johannesburg 1710, South Africa; ntwasmm@unisa.ac.za; Tel.: +27-11-471-2272

**Keywords:** cancer, anticancer drugs, immune checkpoint inhibitors, oncology, Hdm2, Hdm4

## Abstract

Hdm2 and Hdm4 are structural homologs that regulate the tumor suppressor protein, p53. Since some tumors express wild-type p53, Hdm2 and Hdm4 are plausible targets for anticancer drugs, especially in tumors that express wild-type p53. Hdm4 can enhance and antagonize the activity of Tp53, thereby playing a critical role in the regulation of p53’s activity and stability. Moreover, Hdm2 and Hdm4 are overexpressed in many cancers, some expressing wild-type Tp53. Due to experimental evidence suggesting that the activation of wild-type Tp53 can augment the antitumor activity by some checkpoint inhibitors, drugs targeting Hdm2 and Hdm4 may be strong candidates for combining with checkpoint inhibitor immunotherapy. However, other evidence suggests that the overexpression of Hdm2 and Hdm4 may indicate poor response to immune checkpoint inhibitors. These findings require careful examination and scrutiny. In this article, a comprehensive analysis of the Hdm2/Hdm4 partnership will be conducted. Furthermore, this article will address the current progress of drug development regarding molecules that target the Hdm2/Hdm4/Tp53 partnership.

## 1. Introduction

The human p53 gene is involved in almost all cancers, and its regulatory machinery is often dysfunctional in carcinogenesis. The p53 prototypical negative regulator, Mouse Double Minute (Mdm2), is often overexpressed through amplification in cancers carrying a wild-type p53 allele, causing a massive depletion of p53 and aberrant cell proliferation [1,2,3]. Similarly, the other homolog, Mdm4, plays a critical role in the relationship and will be reviewed here [4,5]. A further development is the involvement of p53 in the tumor microenvironment (TME), especially in the function of innate immune cells, which secrete critical cytokines. Moreover, Mdm2 has been identified as a biomarker for resistance to checkpoint inhibitors [6,7]. The current study reviews progress in drug development activities targeting the p53-Mdm2/Mdm4 interface.

This study was conducted by using the Google Scholar search engine using the following terms: p53/mdm2/Mdm4 complex, Mdm2 overexpression, Mdm4 overexpression, and drugs targeting Mdm2/mdm2. The status of drugs was similarly searched through the same search engine, often by adding “clinical trial” to the drug’s name.

The primary aim of this review is to assess the drugability of the p53-Hdm2/Hdm4 interface and the status of current drug development endeavors.

## 2. The Genomic Structure of Hdm2, Hdm4, and p53

The human p53 gene is in chromosome 17p13.1 and is translated into multiple splice isoforms encoding proteins whose specific functions are yet to be comprehensively elucidated. The human Mouse Double Minute (Hdm2) gene in chromosome 12q14.3-q15 encodes a protein with critical functional domains as a negative regulator of p53 together, operating in a feedback loop since Hdm2 is also a p53 transcriptional target [8,9]. These domains include a p53 binding region (p53BD), an acidic region, a Zinc finger motif, and a RING finger domain. The RING domain has E3 ligase activity that is critical for catalyzing the ubiquitination and degradation of the p53 protein by the proteasome. The Hdm4 gene is in chromosome 1q32 and encodes a protein similar in structure to Hdm2, retaining the ability to bind to and inhibit the transactivation of p53 but lacking the E3 ligase activity. Instead, the Hdm4 protein is reported to stabilize the p53-Hdm2 complex and enhance Hdm2 E3 ligase activity by binding to Hdm2 [10,11].

The location of these genes on different chromosomes suggests differences in their regulatory arrangements. Interestingly, the expression of p53 and Hdm2 is regulated by a set of two promoters each, an arrangement conserved in vertebrates and invertebrates. Such a regulatory mechanism could also determine different spatiotemporal expression patterns of these proteins. Extensive studies of the Hdm2 promoters and, to some extent, the Hdm4 promoters, reveal a complex diversity of regulatory configurations [12]. This scenario has critical implications for drug discovery and development since the drugs may target different types of cancer. Therefore, this paper will review the regulatory settings and structure–function relationships of these three genes and their encoded proteins as a background to their functional roles.

**Figure 1:**
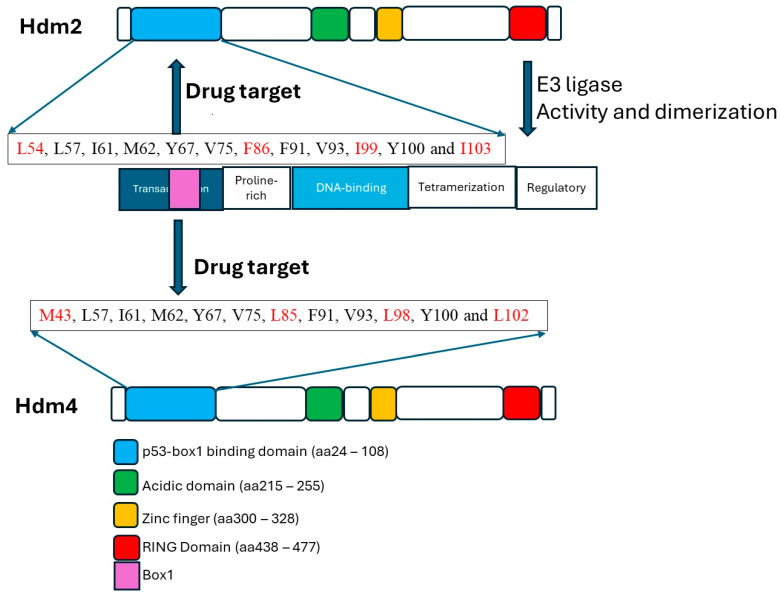
**Structural organization of the Hdm2 and Hdm4 proteins.** The transactivation domain of p53 binds to the p53-binding domains of Hdm2 and Hdm4. This interface contains critical residues in the binding dynamics and is the cornerstone of drug discovery to reactivate p53. These residues presented in the boxes are identical in both proteins except those indicated in red. Even these are mostly conserved in polarity or non-polarity. The RING domain in Hdm2 has E3 ligase activity and is reported to participate in dimerization. The RING domain of Hdm4 does not possess E3 ligase activity but enhances the catalytic activity of Hdm2.

The genomic organization of Hdm2 and Hdm4 genes is identical, except that the Hdm2 promoter region contains p53 response elements. Although on different chromosomes, the conserved genomic structure of Hdm2 and Hdm4, such as conserved intronic-exonic junctions and amino acid residues, suggests a common evolution [13]. Molecular studies from the discovery of Hdm4 have shown that the expression of the transcripts and proteins of p53, Hdm2, and Hdm4 are the same in all tested tissues [13,14]. Under general circumstances, Hdm4 inhibits the transactivation of Hdm2 by binding to the N-terminal domain of p53. However, upon DNA damage and ribosomal stress, Hdm4 preferentially promotes the transactivation of Hdm2 by p53 to other targets, such as p21 [15]. A widely investigated strategy, which has led to innovative drug discoveries, is targeting the p53-Hdm2/Hdm2 interface to reactivate wild-type and mutant p53 (Figure 1).

Although these genes are expressed in all cells, subcellular spatiotemporal expression patterns are reported. For example, Hdm2 is localized in the nucleus in unperturbed cells, whereas Hdm4 is in the cytoplasm [16]. Hdm2^−/−^ and Hdm4^−/−^ mice are embryonic lethal, a phenotype that can be rescued by simultaneous p53 deletion. Furthermore, the E3 ligase activity in Hdm2 is essential, as its deletion is sufficient to produce embryonic lethality [17]. Moreover, the two structural homologs are non-redundant inhibitors of p53 because neither can compensate for the other’s loss during embryonic development. In addition to indicating that they have distinct functions, the evidence shows that, in some instances, they act synergistically to regulate p53 [18]. Notably, heterozygosity does not rescue the *Mdmd2^−/−^* phenotype, but a hypomorphic allele of *p53* lacking the proline-rich domain does not rescue an Hdm2 null phenotype in contrast to Hdm4 [18]. These studies show that Hdm2 and Hdm4 have independent roles in some circumstances and settings.

## 3. Structural Features of the Proteins and Mechanisms of Interaction

To put the drugability of the p53-Hdm2/Hdm4 interface into perspective, dissecting the physiological and chemical molecular interactions in the drug–target binding surface is necessary.

Hdm2 is known to control p53 through two mechanisms. First, through its E3 ligase activity, Hdm2 can polyubiquitinate p53, leading to degradation by the proteasome. Second, a monoubiquitinated p53 will inhibit the transactivation of Hdm2 expression in the nucleus, thereby providing a feedback control mechanism. Hdm4 does not catalyze the degradation of p53 but, instead, abolishes its transcriptional activity [19,20].

Although the p53-binding pockets of Hdm2 and Hdm4 are highly similar, an N-terminally proximal “lid”, considered to possess a substantial regulatory influence on Hdm2-p53 functions, consists of different sequence structures [21]. A comparative analysis of the critical residues in the p53 binding pocket (p53BD) of Hdm2 and Hdm4 indicates subtle differences, yet significant differences exist in their drug interactions (Table 1). This suggests that some of the residues profoundly impact the conformation of the proteins. This binding pocket is critical because it is the target of most drugs designed to reactivate p53 activity by interfering with its interaction with Hdm2 and Hdm4. The findings suggest that Hdm2 and Hdm4 share critical structural features but possess subtle structural differences that may impact their molecular functions. Moreover, Hdm4 was found to exist in various conformations with different abilities to bind p53 [22]. Analyzing the Hdm2/Hdm4/p53 interface is crucial for rational drug design, especially for developing combined drug therapeutic drugs. Consequently, the dual targeting of Hdm2 and Hdm4 interfaces with p53 is attractive for reducing resistance challenges.

A recent comprehensive review of small molecule drugs targeting p53 covers three classes of drugs that target Tp53. These are (i) drugs that block the interaction between p53 and Hdm2, (ii) drugs that correct the conformation of some Tp53 mutants and reactivate normal Tp53 function, and (iii) zinc metallochaperones that act as ionophores that facilitate the binding of zinc Tp53 [23]. The first two types are the subject of this review.

Although the binding interfaces of Hdm2 and Hdm4 on p53 are similar, small molecule drugs such as Nutlin3a have different inhibitory effects. Nutlin3a does not disrupt Hdm4-p53 binding and is a poorer inhibitor of Hdm4 compared to Hdm2 [24,25]. However, knocking Hdm4 down using siRNA reactivates the p53 activity via p21, suggesting a restoration of its transactivation function [24]. Some small molecule drugs that reactivate p53 function act independently of Hdm2 and Hdm4. For example, RITA (reactivation of p53 and induction of tumor cell apoptosis) is genotoxic and acts by refolding the conformation of a mutant p53 [26]. Classes of small-molecule drugs with promising clinical outcomes include imidazolines, spiro-oxindoles, piperidines, quinacridones, and benzodiazepines. Notably, two new drugs need to be developed, including one approved for cardiovascular disease treatment, ezetimibe, a beta-lactam compound of azetidine-2-one class, and alrizomadlin [23,27,28]. Table 2 lists some examples. Some of these drugs have a remarkable impact on outcomes from checkpoint inhibitor treatment, which will be discussed later in the current study.

## 4. Comparative Evaluation of the Chemical Classes

The discovery of a highly potent drug targeting the p53-Hdm2/Hdm4 interface with a Kd < 100 nmoles is a significant scientific achievement and a challenging endeavor.

Advanced drugs targeting the Mdm2/Mdm4–p53 complexes fall in the imidazoline, piperidinone, and Spiro oxindole chemical classes. Imidazolidines, represented by Nutlin-3a or idasanutlin, were the first small-molecule drugs shown to disrupt the interaction between p53 and Hdm2. Nutlins fit into the three hydrophobic pockets of the Mdm2 p53-binding site characterized by Phe19, Trp 23, and Leu26. They stabilize p53 and reactivate the p53 pathway, inducing cell cycle arrest and apoptosis in p53WT cells [29]. Nutlin-3a, which has been extensively studied in clinical settings, proved to have poor pharmacokinetics, although it played critical roles in establishing the concept of disrupting the p53-Mdm2 interaction as a therapeutic strategy. Consequently, it was further developed into RG7112, which has shown an ability to activate the p53 pathway and observable efficacy in solid and hematological tumors after oral administration. RG7112 had an IC_50_ of 0.4 mM by 3-(4,5-dimethylthiazol-2-yl)-2,5-diphenyl-tetrazolium bromide (MTT) compared to Nutlin-3a IC50 of 1.5 mM. By HTRF, RG7112 IC50 was 0.018 mM compared to nutlin-3a IC_50_ of 0.088 mM. Furthermore, the drug selectivity of RG7112 was doubled by modifying nutlin-3a, and the activation of the p53 pathway was observed in this Phase I clinical study. RG7112 also exhibited improved pharmacokinetics in terms of bioavailability and half-life [30]. RG7112 as a monotherapy has been studied in many Phase I trials on hematologic neoplasms, neoplasms, chronic myelogenous leukemia, acute myelogenous leukemia, essential thrombocytopenia, and sarcomas [31]. RG7112 is regarded as the most potent derivative of the original nutlin core. Overall, the RG7112 Phase I clinical study of 116 participants as a monotherapy showed better efficacy and safety, with three dose-limiting toxicities reported [32].

In the latest clinical trial of the Hdm2 Inhibitor in R/R AML for an overall survival (MIRROS) clinical trial, idasanutlin (RG7388) was combined with cytarabine with or without allogeneic hematopoietic stem cell transplant (HSCT) in patients with refractory or relapsed acute myeloid leukemia (R/R AML) [33,34]. Idasanutlin did not have a positive effect on overall survival except when in patients receiving HSCT. Furthermore, increased toxicity was observed in the idasanutlin–cytarabine arm compared to the placebo–cytarabine arm. The MIRROS study was terminated due to futility, and whether idasanutlin has potential in AML and other cancers is still inconclusive. However, a recent study shows that a novel complexing of RG7388 with platinum anticancer drugs produces favorable in vitro and in vivo outcomes [35].

The SJ-172550 drug binds primarily Hdm4 and, to a lesser effect, Hdm2 [36]. This drug was shown to have improved anticancer activity in monotherapy and increased efficacy when combined with erlotinib [37].

The first small molecule inhibitor of the Hdm4/p53 interaction, WK238 (Norvatis-101) binds weakly to p53, probably due to the differences in the residues in the Leu26 pocket (Table 1). WK238 interacts with Hdm2 and Hdm4 with inhibitory constants (K_i_ values) of 109 nmol/l and 11 mmol/l, respectively [38]. In an FP assay, WK238 is reported to have Hdm4 and Hdm2 Ki values of 36 μM and 916 nM, respectively. WK238 was further developed to Norvatis-14 by extensive substitutions to increase potency, producing an analog with TR-FRET IC_50_ of 17 nM [39,40]. The nutlin-3 drug binds to Hdm4 and Hdm2 with Ki values of 70 μM and 69 nM, respectively, even though WK298 appears to have a better binding pose than the nutlin-3a [41]. There are no clinical trial data for either Norvatis-101 or Norvatis-14.

The spiro-oxindoles were designed de novo from a pharmacophore based on the Hdm2 p53-binding pocket, and the lead products were extensively substituted to produce molecules with improved pharmacology, such as M-63, MI-219 (M_-147), and SAR405838. For example, the original lead compound with K_i_ of 86 nM and an IC_50_ of 830 nM was optimized by adding fluorine to the chlorophenyl ring mimicking Phe^19^, producing MI-63 with K_i_ of 3 nM and IC_50_ of 280 nM [42], and activating the wild-type p53 pathway and apoptosis in cancer cell lines but proving unsuitable for pre-clinical studies in vivo. Mi-63 was further developed into MI-219, with 65% oral bioavailability and potent inhibitor activity, and was tested in pre-clinical trials. This compound inhibited cancer cell growth in a wild-type p53-dependent manner because it was ineffective in p53 knock-out cells [43,44]. In a panel of lung cancer cell lines, MI-219 was efficacious and induced G1 and G2 cell arrest. However, MI-219 was shown to act by a mechanism different from that of nutlins, whereby the functional activity of Hdm2 was altered, leading to enhanced autoubiquitination and degradation [45]. The MI series is still under development at Ascenta and the University of Michigan. It is potent and has a remarkable affinity. A derivative of MI-219, called SAR405838, was developed by Sanofi-Aventis and bound to Hdm2 with substantially improved affinity and selectivity. Moreover, SAR405838 activated wild-type 953 at nanomolar concentrations [46].

A Phase I study of SAR405838 was recently conducted and showed an acceptable safety profile [47]. The dose escalation study enlisted patients with locally advanced metastatic solid tumors. In flank xenografts, SAR405838 was efficacious but not in orthotopic tumors. Efficacy in orthotopic tumors was improved by enhanced drug delivery in glioblastoma, indicating that specialized delivery is required when the blood-brain barrier is compromised [48]. When SAR405838 was combined with an MEK inhibitor, rigosertib, to determine efficacy and pharmacokinetics, the best overall response was stable disease (SD), and the prolonged SD (>six months) was observed with colorectal cancer patients. This result indicated the feasibility of reactivating p53 while inhibiting the MAP kinase pathway to improve anticancer treatment [49]. A pre-clinical study on xenograft models of liposarcoma shows that oral administration of SAR405838 archives good bioavailability and confirms the pharmacodynamic effects associated with the activation of p53 [50]. Therefore, recent studies indicate that spiro-oxindole has excellent potential to develop into effective anticancer therapies.

**Table 2:** Classes of drugs targeting Hdm2/Hdm4/p53 complex. * indicates that the drug is a dual inhibitor of Hdm2/p53 and Hdm4/p53 interactions. ↓ indicates a repurposed drug.

Class	Drug Candidates	MoA	Comments	References
Imidazolidines/pyrrolidines	Nutlin3a (idasanutlin), also known as RG7388, RG7112	Mimic p53 Box1 peptide	Advanced to Phase III. Enhanced potency, selectivity, and bioavailability. Solid and hematological tumors. Nutlin3a is a poor antagonist of Hdm4. Severe side effects led to the abandonment of Phase III clinical trials due to futility.	[25,29,33,51,52,53]
	WK298 * (also known as Novartis-101)	Inhibits Hdm4-p53 interaction. Binds both Hdm2 and Hdm4	Hdm4 inhibitor.	[54,55]
Pyrazolylidine	SJ-172550 *	Prevents the formation of p53-Hdm4 complex by changing the conformation of Hdm4	Pre-clinical. Chemically and thermally stable. Needs further optimization.	[22,36]
Spiro-oxindoles	MI-63		Poor PK and poor oral bioavailability.	[54]
	MI-219		Poor PK and improved oral bioavailability and minimal toxicity in mice.	[54]
	MI-147		Highly potent, highly desirable PK, better oral bioavailability than MI-219, and low toxicity.	[56]
	MI-188		Highly potent with excellent oral PK profile and bioavailability, efficacious with minimal toxicity.	[56]
	MI-1061		Good oral bioavailability and chemical stability in mice.	[57]
Piperidinone	AMG-232 (navtemadlin)	Inhibits p53-Hdm2 interaction	Phase I orally bioavailable.	[58,59]
Stapled peptides (peptidomimetic)	ALRN-6924 *	Hdm2. Mimics the a-helical peptide N-terminal peptide of p53 and binds with high affinity to Mdm2 and Mdm4	Phase I solid and hematological cancers.	[60]
Benzodiazepines	TDP521252	Inhibit p53-Hdm2 complex		[61]
	TDP665759	Inhibit p53-Hdm2 complex		[61]
New drugs Azetidine-2-one	↓ Ezetimibe	Inhibits p53-Hdm2	Pre-clinical.	[27,28]
Spiro-oxindole	APG-115 ( alrizomadlin)			[62,63,64]
	SAR405838 (MI-773)		Phase I. Stable with favorable PK in mice, rats, and dogs.	

## 5. The Potential p53 Influence on Checkpoint Inhibitors

The tumor microenvironment (TME) consists of normal stromal cells, tumor-associated stromal cells, tumor-associated immune cells, endothelial cells, and cancer stem cells CSCs). The primary role of the tumor suppressor protein, p53, appears to be protection against cancer progression and is expressed endogenously in bone marrow-derived macrophages. It has been demonstrated that p53 plays a critical role in modulating the TME by influencing classically activated macrophages (MI) and alternatively activated (M2) macrophage polarization, whereby p53 deletion or activation profoundly impacts tumorigenesis. The M1 state is tumor-inhibiting, while the M2 state is tumor-promoting. The expression of M2 genes is increased in p53-deficient and p53-mutant mice. Moreover, p53-deficient cells promote tumorigenesis non-cell-autonomously. A study in an adenoma-prone Apc^Min/+^ mouse model showed that a loss of one allele of p53 resulted in the initiation of adenomas and excessive tumor burden in the intestines of these mice [65,66,67]. These findings suggest a profound impact that p53 exerts on the phenotype of TME cells regardless of their genotype.

It is established that chemokines play critical roles in tumorigenesis, such as influencing anti-tumor immune response and angiogenesis, controlling tumor infiltrating by innate immune cells, and metastasis. In the TME, immune, tumor, and stromal cells are signaled by autocrine and paracrine growth factors, which are critical for tumorigenesis [68,69]. This review assesses the impact of p53 on the tumor environment primarily by looking at the relationship between chemokine signaling and p53 status.

Evidence shows that p53 plays critical roles in the tumor microenvironment, including negative regulation of inflammatory response and cancer stem cell (CSC) generation [70]. In the TME, the dysregulation of p53 function contributes to immune evasion and cancer progression. P53 influences the functions of the Major Histocompatibility Complex class 1 (MHC-I), affecting immune recognition. It also affects the Programmed Death Ligand 1 (PDL1-1), influencing the regulation of a critical immunosuppressive molecule [71,72]. Moreover, p53 is regulated by cytokine signaling, such as type I interferons (IFNs), interleukin-6 (IL-6), and macrophage migration inhibitory factors, exerting significant influence in inflammatory immune response associated with tumorigenesis [6,73,74]. These observations indicate that anticancer drug development targeting the p53 pathway activities in the TME is plausible.

An emerging concern about the use of immune checkpoint inhibitors (ICIs) is the occurrence of tumor hyper-progression. Hdm2 and Hdm4 amplification have been proposed as a possible biomarker of this phenomenon [7,75]. Identifying these prototypical negative regulators of p53 as potential biomarkers for tumor hyper-progression after immunotherapy underlines the importance of proper elucidation of the p53 influence on the TME.

In 2017, a new drug, Alrizomadlin (APG-115), was found to have antitumor activity in the TME. Alrizomadlin is a spiro-oxindole that binds with high affinity to Hdm2 and has good oral pharmacokinetics [62]. Alrizomadlin was found to stimulate the reduction of the immunosuppressive M2 macrophages, the increase in M1 macrophages, and the expression of PD-L1 in tumor cells. Moreover, alrizomadlin acted synergistically in syngeneic mouse models in *Tp53^wt^*, *Tp53^−/−^*, and *Trp^mut^* backgrounds, and this effect was abolished when *Tp53* was deleted [64]. This is important because it shows that alrizomadlin reactivates p53 in the TME and causes tumor reduction regardless of the tumor p53 status. A Phase II study of alrizomadlin combined with the PD-1 inhibitor, pembrolizumab, indicates that the combination is well tolerated, with prospects for alternative treatment in cancers that resist immuno-oncology drugs [76].

## 6. Challenges and Future Directions

The toxicity of drugs targeting the p53-hdm2/hdm4 interface is a critical challenge. One of the probable causes of toxicity emanates from the rationale behind the p53 reactivation strategy. Since a feedback loop characterizes the p53-Hdm2 interaction, preventing p53 ubiquitination could result in the accumulation of Hdm2, without the ability to degrade the active p53 through ubiquitination. Therefore, the challenge is balancing the resultant accumulation of Hdm2 and the reactivation of p53. A resolution of the dilemma could assist in reducing toxicity. Another layer of research that could be explored is the targeted delivery of these drugs through nanotechnology. This might be useful in treating some solid cancers.

## 7. Conclusions

Several drugs targeting the p53-Hdm2/Hdm4 interface are under development, but they have yet to progress past clinical stages due to acquired drug resistance, dose-dependent toxicity, and poor efficiency. The most extensively investigated drugs belong to the imidazolidine/pyrrolidine class, including nutlin-3a, which proceeded to Phase III clinical trial in combination with chemotherapy. The remaining drugs are in early clinical studies and show promising pharmacokinetics but must pass the crucial aspect of toxicity. Furthermore, the drugs must be effective alone and with other therapies. It is encouraging that the majority exhibit good oral bioavailability. The emerging approach to administering these drugs in combination with checkpoint inhibitors is biologically rational and promising, given the outcomes observed with alrizomadlin.

## Figures and Tables

**Table 1:** Critical residues in the Hdm2/Hdm4 pocket and the p53TAD. + denotes identical residues. Asterisks indicate interacting residues with the three critical binding pockets.

Hdm2 Residues	Hdm4	p53 Transactivation Domain Residues	Comments
L54 **	**M43**	F19 * (binding pocket)	L54 forms hydrogen bond with p53 W23
L57	+	W23 ** (binding pocket)	
I61	+	N29 ***	
M62	+	L26 (binding pocket)	
Y67	+		
Q72 *	+		Forms hydrogen bond with p53 F19
V75	+		
F86	L85		
F91	+		
V93	+		
I99	L98		
Y100 ***	+		Forms hydrogen bond with p53 N29
I103	L102		

The number of asterisks indicates interacting partners between column 1 and column 3.

## Data Availability

Not applicable.
